# Different patient and activity-related characteristics result in different injury profiles for patients with anterior cruciate ligament and posterior cruciate ligament injuries

**DOI:** 10.1007/s00167-022-07131-y

**Published:** 2022-08-27

**Authors:** Janina Kaarre, Bálint Zsidai, Philipp W. Winkler, Eric Narup, Alexandra Horvath, Eleonor Svantesson, Eric Hamrin Senorski, Volker Musahl, Kristian Samuelsson

**Affiliations:** 1 Department of Orthopaedics, Institute of Clinical Sciences, Sahlgrenska Academy, University of Gothenburg, Göteborgsvägen 31, 431 80 Mölndal, Sweden; 2 Sahlgrenska Sports Medicine Center (SSMC), Gothenburg, Sweden; 3 Department of Orthopaedic Surgery, UPMC Freddie Fu Sports Medicine Center, University of Pittsburgh, Pittsburgh, USA; 4 Department of Sports Orthopaedics, Klinikum Rechts Der Isar, Technical University of Munich, Munich, Germany; 5 Department of Orthopaedics and Traumatology, Kepler University Hospital Linz, Linz, Austria; 6 Department of Internal Medicine and Clinical Nutrition, Institute of Medicine, Sahlgrenska Academy, University of Gothenburg, Gothenburg, Sweden; 7 Unit of Physiotherapy, Department of Health and Rehabilitation, Institute of Neuroscience and Physiology, Sahlgrenska Academy, University of Gothenburg, Gothenburg, Sweden; 8 Department of Orthopaedics, Sahlgrenska University Hospital, Mölndal, Sweden

**Keywords:** Injury profile, Cruciate ligament injuries, Anterior cruciate ligament, ACL, Posterior cruciate ligament, PCL reconstruction

## Abstract

**Purpose:**

To compare patient characteristics including patient sex, age, body mass index (BMI), activities at the time of injury and injury profiles in patients with anterior cruciate ligament (ACL) and posterior cruciate ligament (PCL) injuries.

**Methods:**

Data were obtained from the Swedish National Knee Ligament Registry. Two study groups were created: (1) index ACL reconstruction (ACL group) and (2) index PCL reconstruction (PCL group). Between-group differences were investigated using Fisher’s exact test and Fisher’s non-parametric permutation test for dichotomous variables and continuous variables, respectively.

**Results:**

Of 39,010 patients, 38,904 were ACL injuries. A larger proportion of patients with combined injuries to the PCL, meniscus and cartilage were female, aged > 25 years and with a BMI of > 35 kg/m^2^ compared with patients with combined injuries to the ACL, meniscus and cartilage. An isolated ACL injury was more commonly found in males, while all other injury profiles of ACL, including combined injuries with meniscus, cartilage and collateral ligament injuries, were more frequently observed in females. The PCL injuries were sustained either during pivoting sports, non-pivoting sports or were traffic-related.

**Conclusion:**

Different patient characteristics (BMI, age and sex), and activities at the time of injury (sport- versus traffic-related activities), resulted in distinct injury profiles for the ACL and PCL groups. These findings provide valuable information of the way specific injury patterns of cruciate ligament injuries occur, and subsequently may help clinicians with the diagnostic process of ACL and PCL injuries.

**Level of evidence:**

III.

## Introduction

Injuries to the cruciate ligaments, including anterior cruciate ligament (ACL) and posterior cruciate ligament (PCL) tears, are severe injuries affecting athletes in their ability to return to sport [23, 25, 26]. Previous studies have demonstrated a higher incidence of PCL injuries in men [14, 17], while female athletes appear to run a greater risk of sustaining an ACL injury [4, 11]. Furthermore, ACL injuries are reported to occur more commonly in young high-level female athletes, while males affected by an ACL injury are reported to be somewhat older [24]. A higher body mass index (BMI) has also been related to an increased risk of sustaining cruciate ligament injuries[29] and has been reported to be an important contributory factor in combined knee ligament injuries [28]. Additionally, different injury profiles of knee ligament injuries have been linked to different types of injury mechanism, where ACL injuries have usually been associated with non-contact sports and PCL injuries with high-energy trauma [6, 14, 20, 33]. Consequently, PCL injuries more frequently coincide with additional knee ligament tears, while ACL injuries more often occur with concurrent meniscal lesions [20, 30].

Despite the rising incidence of cruciate ligament injuries and their negative impact on both sports participation and general knee function, the specific injury profiles for ACL and PCL injuries are yet to be described. Thus, the purpose of this study was to explore and compare patient characteristics including patient sex, age, BMI and activity at the time of injury, as well as injury profiles in patients undergoing either ACL or PCL reconstruction. It was hypothesised that ACL and PCL injuries would have distinct injury profiles, where ACL injuries would be more common in younger patients with a lower BMI, while patients with PCL injuries would be older and have a higher BMI. A deeper understanding of the profiles of these severe knee injuries could lead to improved treatment planning and, subsequently, improved treatment-related outcomes. Additionally, an increased knowledge of injury profiles for cruciate ligament injuries could provide essential information relating to possible factors associated with specific forms of ACL and PCL injury in order to formulate secondary prevention strategies based on patients’ individual characteristics and activities.

## Materials and methods

This retrospective cohort study was approved by the Swedish Ethical Review Authority (registration number: 2020-03559 and 2021-01002) and was performed in accordance with the Declaration of Helsinki.

Data were obtained from the Swedish National Knee Ligament Register (SNKLR), which mainly collects data on surgically treated ACL and PCL injuries. The registry includes both surgeon- and patient-reported information, including demographical characteristics, injury- and surgery-related factors and patient-reported outcome measurements (PROMs) [2]. Participation in the SNKLR is optional and exclusion can be requested if research participation is not desired by the patient. The registry has previously been described in more detail [12].

### Data collection and study sample

The data obtained from the SNKLR were collected between 1 January 2005 and 31 December 2019. Only data on patients undergoing either ACL reconstruction (ACL-R) or PCL reconstruction (PCL-R) were obtained from the register. Two main study groups were created, including patients who had undergone an index ACL reconstruction (ACL group) and an index PCL reconstruction (PCL group). Additionally, the following groups were created for subgroup analysis: isolated ACL injury, ACL injury + meniscal injury + cartilage injury, ACL injury + collateral ligament injury, isolated PCL injury, PCL injury + meniscal injury + cartilage injury and PCL injury + collateral ligament injury. Patients with any concomitant fracture, as well as combined ACL and PCL injuries, were excluded. In addition, patients with revision ACL or PCL, or previous knee surgeries were excluded from further assessment. Patient characteristics including age, patient sex, BMI, smoking status, activity at the time of injury and the type of injury (ACL versus PCL injuries) were extracted from the SNKLR. Surgical data on concomitant injuries, including information on neurovascular injuries, injuries to the posterior lateral corner (PLC) and cartilage lesions, as well as meniscal and collateral ligament injuries, were extracted for further analyses. Activities at the time of injury were divided into the following categories: sports-related injuries, including alpine/skiing, pivoting sport, non-pivoting sport and other physical activity (other recreational sport, exercise and trampoline), as well as traffic-related injuries and other activities at the time of injury (other, outdoor and work activity). Moreover, sports including American football/rugby, basketball, dancing, floorball, gymnastics, handball, ice hockey, bandy, martial arts, racket sports, football, volleyball and wrestling were categorised as pivoting sports, while cross-country skiing, cycling, horseback riding, motocross/enduro, skateboarding, snowboarding and surfing/wakeboarding were categorised as non-pivoting sports.

### Statistical analyses

The statistical analyses were performed using the SAS System for Windows (version 9, SAS Institute, North Carolina, USA). Continuous and ordinal data are presented as the mean and standard deviation (SD), as well as the median with minimum and maximum, while count (*n*) and proportion (%) are used for dichotomous variables. Between-group differences were investigated using Fisher’s exact test for dichotomous variables. Fisher’s non-parametric permutation test was used to compare differences in continuous variables between the study groups. All the tests were two-tailed at a 5% significance level.

## Results

**Fig. 1:**
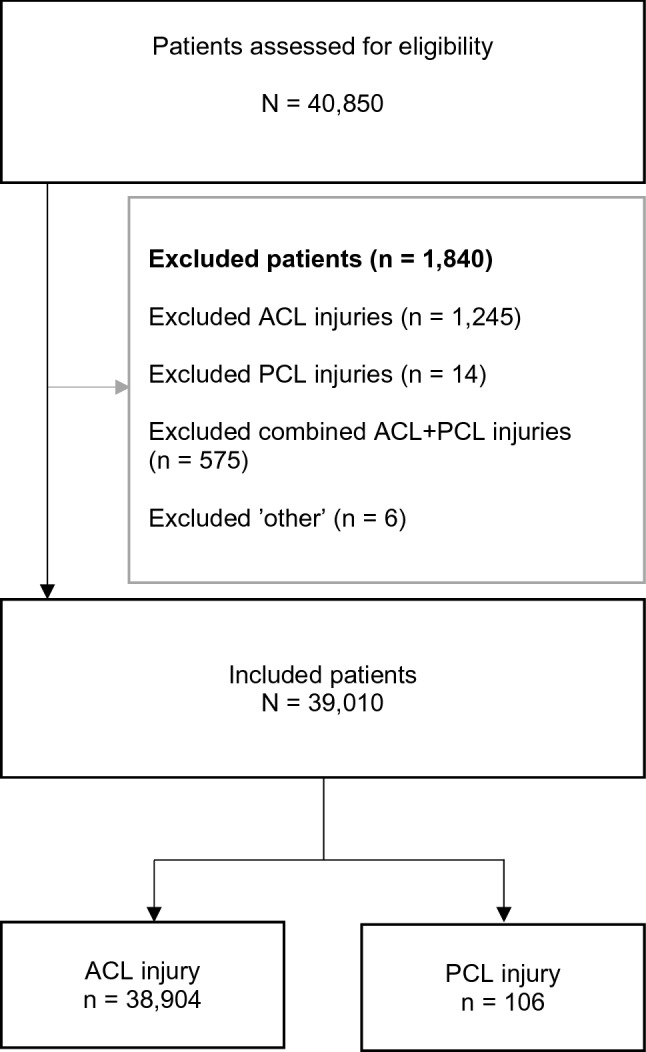
Flow chart of patient enrolment. Values are given as the count (*n*). *ACL* anterior cruciate ligament, *PCL* posterior cruciate ligament

Baseline data were available for 39,010 patients (Fig. 1), of which 38,904 patients had undergone an ACL reconstruction. The ACL group was significantly younger compared with the PCL group (mean 27 ± 10 and 31 ± 13 years), *p* < 0.01). Male sex was reported for 57% of patients with ACL-R and 62% of patients with PCL-R (*p* = 0.32). There was no significant difference between groups with regard to BMI (*p* = 0.15). Table 1 presents a detailed description of patient characteristics.

**Table 1:** Baseline characteristics of patients with either an ACL or PCL injury

Variable	Total (*n* = 39,010)	ACL (*n* = 38,904)	PCL (*n* = 106)	*P*
Males, *n* (%)	22,247 (57.0)	22,181 (57.0)	66 (62.3)	n.s
Age (years)	27.2 ± 10.2 25 (7–74)	27.2 ± 10.2 25 (7–74)	30.8 ± 13.0 26.5 (16–69)	** < 0.01**
BMI (kg/m^2^)	24.6 ± 3.6 24.2 (14.9–65.8)	24.6 ± 3.6 24.2 (14.9–65.8)	25.4 ± 4.3 24.4 (19.7–36.5)	n.s
Smoking, *n* (%)	1,092 (5.5)	1,089 (5.5)	3 (7.3)	n.s
Right knee, *n* (%)	20,272 (52.0)	20,235 (52.0)	37 (34.9)	** < 0.001**
**Activity at time of injury**				
**Sports related, *n* (%)**	33,000 (84.8)	32,944 (84.9)	56 (52.8)	** < 0.001**
Alpine/skiing, *n* (%)	6,227 (16.0)	6,219 (16.0)	8 (7.5)	**0.02**
Pivoting sport, *n* (%)	25,073 (64.4)	25,039 (64.5)	34 (32.1)	** < 0.001**
Non-pivoting sport, *n* (%)	1,700 (4.4)	1,686 (4.3)	14 (13.2)	** < 0.001**
Other physical activity, *n* (%)	1,524 (3.9)	1,516 (3.9)	8 (7.5)	n.s
**Traffic related, *n* (%)**	783 (2.0)	758 (2.0)	25 (23.6)	** < 0.001**
**Other, *n* (%)**	3,607 (9.3)	3,590 (9.3)	17 (16.0)	**0.04**

Values are given as *n* (%) and the mean (SD)/median (minimum–maximum) for categorical variables and continuous variables respectively

For comparisons between groups, Fisher’s exact (lowest 1-sided *p*-value multiplied by 2) and Fisher’s non-parametric permutation tests were used for dichotomous variables and continuous variables respectively

*ACL* anterior cruciate ligament, *BMI* body mass index, *PCL* posterior cruciate ligament, *SD* standard deviation

Pivoting sport: American football/rugby, basketball, dancing, floorball, gymnastics, handball, ice hockey/bandy, martial arts, racket sports, football, volleyball, wrestling. Non-pivoting sport: cross-country skiing, cycling, horseback riding, motocross/enduro, skateboarding, snowboarding and surfing/wakeboarding. Other physical activity: other recreational sport, exercise, trampoline. Other: other, outdoor activity and work

### Concomitant injuries

The presence of concomitant injuries compared between the ACL group and PCL group did not reach statistical significance (*p* = 0.05; Table 2). However, more cartilage injuries were seen in the PCL group (38%) compared with the ACL group (26%) (*p* = 0.01) and ACL injuries more frequently occurred in conjunction with meniscal injuries (44%) compared with PCL injuries (23%) (*p* < 0.001). Furthermore, concomitant collateral ligament injuries were more frequently present in the PCL group (47%) compared with the ACL group (5.0%) (*p* < 0.001; Table 2).

**Table 2:** Concomitant injuries for patients with either an ACL or PCL injury

Variable	Total (*n* = 39,010)	ACL (*n* = 38,904)	PCL (*n* = 106)	*P*
Concomitant injury, *n* (%)	22,289 (57.1)	22,218 (57.1)	71 (67.0)	n.s
**Meniscal injury, *n* (%)**	17,054 (43.7)	17,030 (43.8)	24 (22.6)	** < 0.001**
Lateral meniscus injury, *n* (%)	9,539 (24.5)	9,524 (24.5)	15 (14.2)	**0.01**
Medial meniscus injury, *n* (%)	10,299 (26.4)	10,287 (26.4)	12 (11.3)	** < 0.001**
**Cartilage injury, *n* (%)**	10,276 (26.3)	10,236 (26.3)	40 (37.7)	**0.01**
Lateral femoral condyle, *n* (%)	1,097 (52.4)	1,092 (52.5)	5 (38.5)	n.s
Medial femoral condyle, *n* (%)	4,566 (65.3)	4,547 (65.3)	19 (67.9)	n.s
Lateral patella, *n* (%)	648 (60.0)	642 (60.1)	6 (54.5)	n.s
Medial patella, *n* (%)	1,049 (56.1)	1,040 (56.0)	9 (69.2)	n.s
Lateral tibial plateau, *n* (%)	1,292 (53.3)	1,284 (53.3)	8 (53.3)	n.s
Medial tibial plateau, *n* (%)	1,006 (49.3)	1,000 (49.4)	6 (42.9)	n.s
Trochlea, *n* (%)	646 (56.3)	640 (56.3)	6 (54.5)	n.s
**Neurovascular injury**, ***n* (%)**	27 (0.1)	23 (0.1)	4 (3.8)	** < 0.001**
**Collateral ligament injury (LCL, MCL)**, ***n* (%)**	1,996 (5.1)	1,946 (5.0)	50 (47.2)	** < 0.001**
LCL, *n* (%)	446 (1.1)	419 (1.1)	27 (25.5)	** < 0.001**
MCL, *n* (%)	1,603 (4.1)	1,576 (4.1)	27 (25.5)	** < 0.001**
**PLC, *n* (%)**	135 (0.3)	112 (0.3)	23 (21.7)	** < 0.001**

Values are given as n (%) and the mean (SD)/median (minimum–maximum) for categorical variables and continuous variables respectively

For comparisons between groups, Fisher’s exact (lowest 1-sided p-value multiplied by 2) and Fisher’s non-parametric permutation tests were used for dichotomous variables and continuous variables, respectively

*ACL* anterior cruciate ligament, *LCL* lateral collateral ligament, *MCL* medial collateral ligament, *PCL* posterior cruciate ligament, *PLC* posterior lateral corner, *SD* standard deviation

### Activity at time of injury

The most frequently reported activity at the time of injury for both the ACL and PCL groups was sports. However, a larger proportion of ACL injuries were sustained during sports than for PCL injuries (*p* < 0.001). Among sports-related ACL injuries, 65% and 16% were associated with pivoting sports and alpine/skiing respectively, while sports-related PCL injuries were most frequently caused by pivoting (32%) and non-pivoting sports (13%). Moreover, a larger proportion of traffic-related injuries was reported among patients with PCL tears (24%) compared with the ACL group (2.0%, *p* < 0.001; Table 1).

### Injury profile

**Fig. 2:**
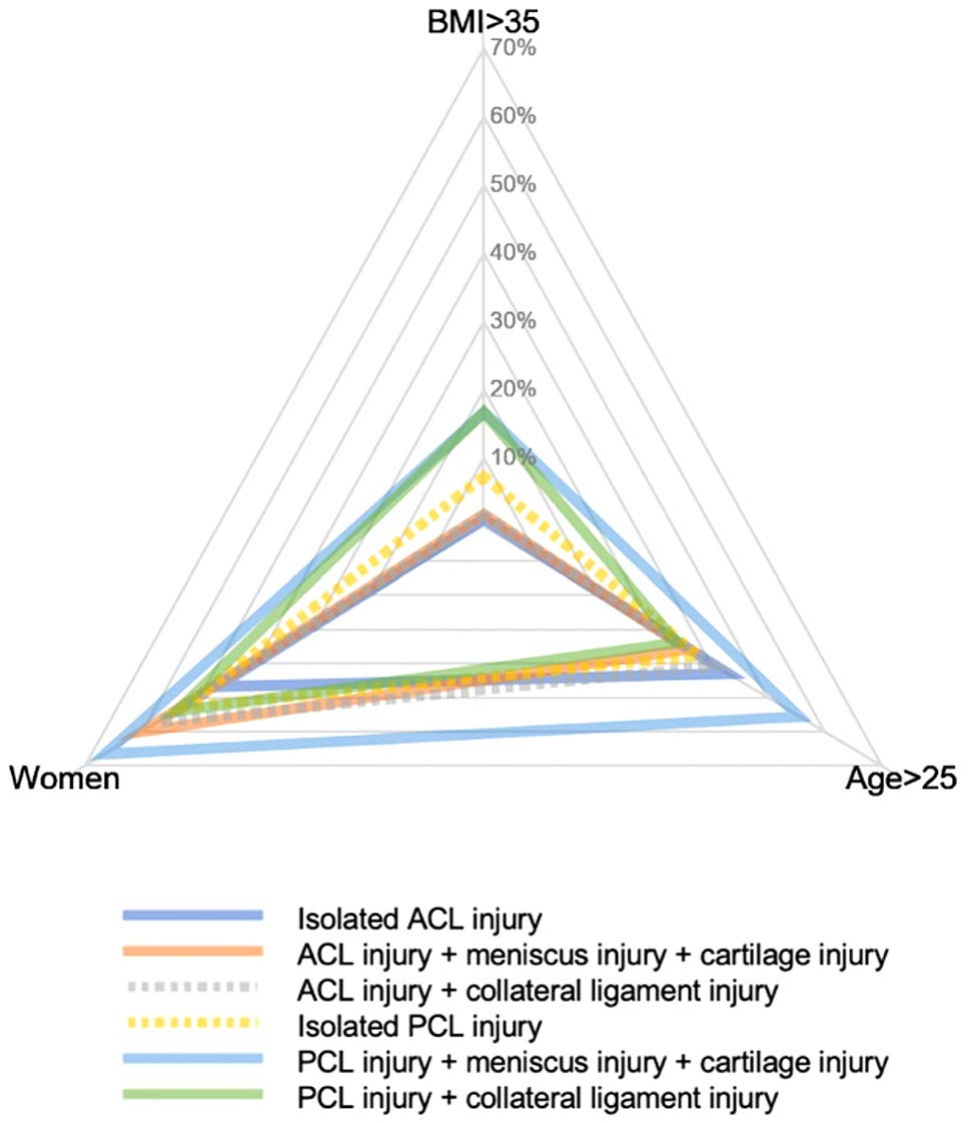
Spider chart of different injury profiles, by demographical dimension. Different patient characteristics are found in different injury patterns for ACL and PCL injuries. *ACL* anterior cruciate ligament, *BMI* body mass index, *PCL* posterior cruciate ligament

Different patient characteristics resulted in distinct injury profiles for ACL and PCL injuries (Fig. 2.). Patients with combined injuries to the PCL, meniscus and cartilage were more often female, aged > 25 years and with a BMI of > 35 kg/m^2^ compared with patients with combined injuries to the ACL, meniscus and cartilage. Moreover, patients with combined injuries to the PCL and collateral ligament had a higher BMI (BMI > 35 kg/m^2^) and they were slightly younger and more frequently male compared with patients with combined injuries to the PCL, meniscus and cartilage. Patients with ACL injuries generally had a lower BMI (BMI < 35 kg/m^2^) compared with patients with PCL injuries. An isolated ACL injury was more commonly found in males, while all other injury profiles of ACL, including combined injuries with meniscus, cartilage and collateral ligament injuries, were more frequently observed in females. Additionally, a larger proportion of PCL injuries was found in females compared with males.

**Fig. 3:**
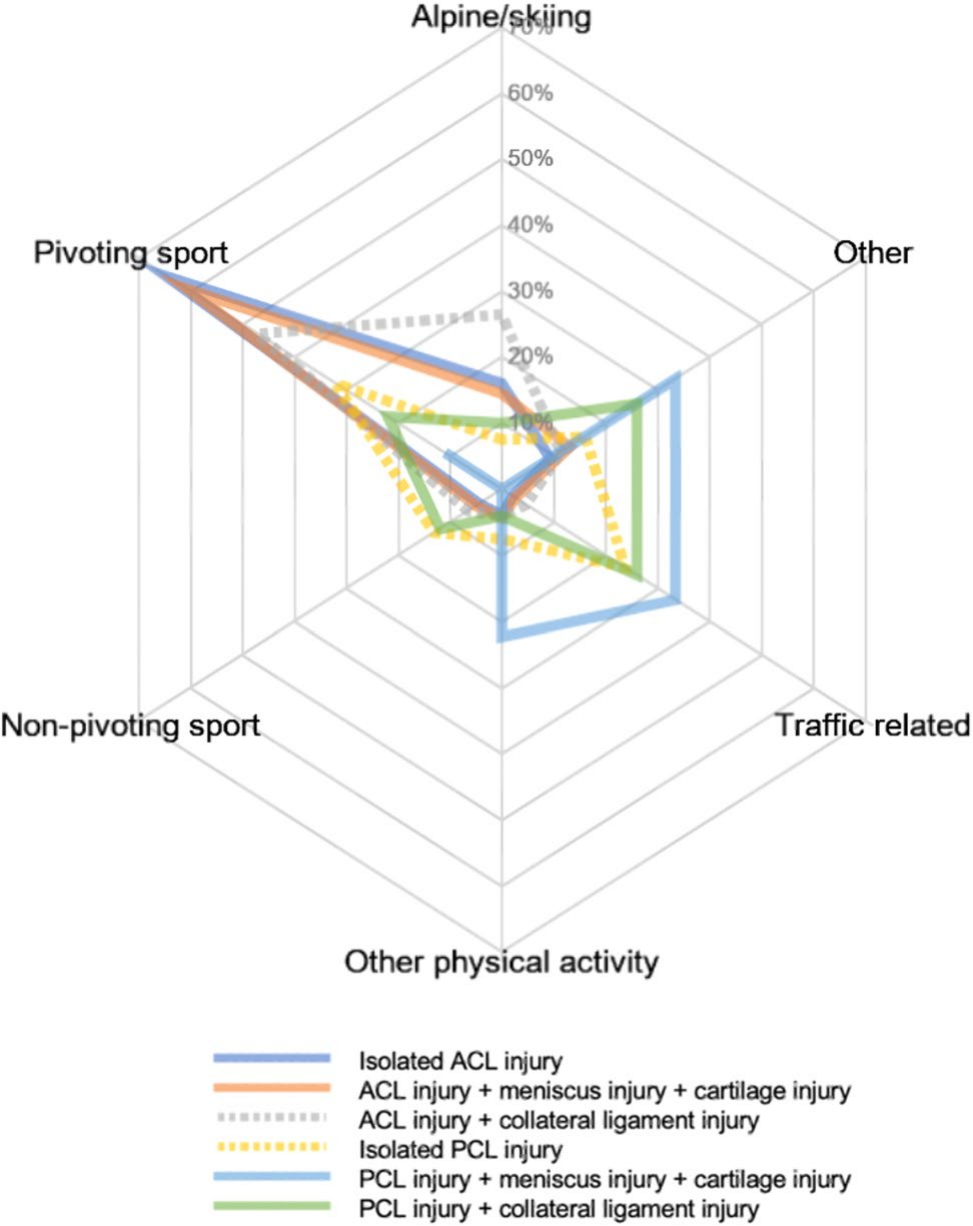
Spider chart of different injury profiles, by the type of activity at the time of injury. Differences in activity at the time of injury are found among the specific injury profiles of ACL- and PCL-injured knees. Pivoting sport: American football/rugby, basketball, dancing, floorball, gymnastics, handball, ice hockey/bandy, martial arts, racket sports, football, volleyball, wrestling. Non-pivoting sport: cross-country skiing, cycling, horseback riding, motocross/enduro, skateboarding, snowboarding and surfing/wakeboarding. Other physical activity: other recreational sport, exercise, trampoline. Other: other, outdoor activity and work. *ACL* anterior cruciate ligament, *PCL* posterior cruciate ligament

Differences in activities at the time of injury were identified among the specific injury profiles of ACL and PCL injury groups (Fig. 3). Pivoting sport was the most prevalent activity at the time of injury in patients with an isolated ACL injury, while an isolated PCL injury was the result of either pivoting sports, non-pivoting or traffic-related activity. Moreover, combined injuries to the ACL, meniscus and cartilage were most frequently the result of a pivoting sport, while both alpine/skiing and pivoting sport were the most common activities at the time of injury for a combined injury to the ACL and collateral ligaments. Both an isolated PCL injury and a combined injury to the PCL and collateral ligaments were usually either traffic related or occurred during pivoting sport. However, a PCL injury combined with meniscal and cartilage lesions was most frequently sustained in a traffic-related activity at the time of injury or other physical activities.

## Discussion

The most important findings in this study were the differences in injury profiles for patients with ACL and PCL injuries. An isolated ACL injury was more commonly found in males, while all other injury profiles of ACL, including combined injuries with meniscus, cartilage and collateral ligament injuries, were more frequently observed in females compared with males. Moreover, a larger proportion of PCL injuries was found in females compared with males.

Concomitant injuries to the collateral ligaments and the articular cartilage were more prevalent in the PCL group compared with the ACL group. In contrast, ACL injuries more frequently occurred either in isolation or in combination with meniscal injuries. These findings are in agreement with previous studies reporting a higher incidence of various concomitant injuries in a population with PCL injuries, whereas ACL injuries tend to occur either in isolation or in conjunction with meniscal lesions [8, 24]. Furthermore, the higher occurrence of concomitant injuries in PCL-injured populations has previously been attributed to high-energy injury mechanisms, leading to more complex patterns of knee ligament injuries [7, 8, 35]. In line with previous research, [3, 8, 9, 27], a high rate of traffic- and sports-related injury mechanisms has been reported in the population with a PCL injury, while ACL injuries most commonly occurred due to sports-related activities, especially pivoting sports.

Further differences in patient characteristics were noted between injury profiles of ACL and PCL injuries. A larger proportion of patients with combined injuries to the PCL, meniscus and cartilage was more frequently female, slightly older (> 25 years) and had a higher BMI (> 35 kg/m^2^) compared with the patients with combined injuries to the ACL, meniscus and cartilage. However, the high rate of PCL injuries combined with meniscus and cartilage lesions in female patients with a higher BMI may be explained by the knowledge that females more commonly sustain knee ligament injuries due to lower-energy mechanisms compared with males who are more frequently subject to high-energy injuries [33]. As a result, this information regarding female sex, a higher BMI and the relationship of these characteristics with complex PCL injuries may be helpful during the diagnostic process in order to arrive at a correct diagnosis, as well as being able to provide accurate treatment. Additionally, an increased BMI (≥ 30 kg/m^2^) has previously been associated with an increased risk of serious knee ligament injuries following low-energy trauma [22].

Isolated ACL injuries were more prevalent in males, while all other injury profiles of ACL and PCL injuries were more frequently seen in females. It has previously been established that female athletes run a higher risk of sustaining an ACL injury compared with males [4, 21, 34], resulting in a greater possibility of different injury profiles and concomitant injuries. Moreover, female athletes have been reported to have a higher BMI compared with males, causing higher ground reaction forces[31] and subsequently increasing the risk of more complex knee injuries. This finding may be of prognostic value following injury, since isolated ACL reconstruction can lead to superior outcomes and a lower rate of long-term osteoarthritis compared with ACL reconstruction with concomitant injuries [10].

Differences with regard to activity at the time of injury were identified among the specific injury profiles for ACL and PCL injuries and are in partial agreement with previous studies [14, 32, 35]. While pivoting sport was the most prevalent activity at the time of injury in patients with isolated ACL injuries, an isolated PCL injury was the result of either pivoting sports, non-pivoting or traffic-related activity. Although the high rate of ACL injuries due to pivoting sports is well established [9, 15], the relationship between PCL injuries and non-pivoting sports is less well known. However, the higher prevalence of PCL injuries compared with ACL injuries in non-pivoting athletes could be explained by the relatively greater popularity of cross-country skiing and snowboarding in Sweden compared with other countries, where pivoting sports including football and basketball are more popular Furthermore, ACL injuries accompanied by meniscus and cartilage injuries were most frequently observed in conjunction with pivoting sports activity at the time of injury, while alpine/skiing and pivoting sport were the most prevalent mechanisms of injury for ACL injuries with collateral ligament injuries. Additionally, the high incidence of combined ACL/MCL injuries in the alpine/skiing population has previously been reported[18, 19] and may be attributed to the high-energy impact and forces usually seen in skiing injury situations. Furthermore, pivoting sport activities have previously been associated with combined injuries to the ACL and meniscus [3, 5], supporting the importance of the meniscus as a secondary stabiliser during pivoting activities [13, 16]. Interestingly, isolated PCL and combined PCL and collateral ligament injuries were caused by similar activities and, as a result, no large differences in activities at the time of injury were observed. Since the activity at the time of injury is similar in these groups, patient characteristics, such as a higher BMI, may possibly be an explanation of the observed difference in injury profiles. Conversely, PCL injuries combined with meniscal and cartilage lesions were most commonly the result of traffic-related injury mechanisms or other physical activities, demonstrating an association between high-energy trauma and more complex knee ligament injuries.

This study has several strengths and limitations. The strengths in the current study include the overall large sample size, including detailed information on a total of 39,010 patients. Additionally, the SNKLR has been estimated to cover approximately 90% of all ACL reconstructions in Sweden [1, 2] and the study sample is therefore representative of the Swedish ACL-reconstructed population. One limitation of this study was the relatively small sample size of included PCL injuries. The primary focus of the SNKLR is the collection of data with regard to surgically treated ACL injuries, which can, in turn, explain the small sample size of patients with PCL injuries. In addition, the annual incidence of PCL injuries is considerably lower compared with the annual incidence of ACL injuries [24, 25], which can partly explain the small sample size of included PCL injuries. Finally, this study was retrospective in nature, which can only indicate relationships between patient characteristics and ACL or PCL injury profiles rather than causal relationships. However, the results of this study, demonstrating distinct injury profiles for ACL and PCL injuries, can be considered as clinically important and subsequently, may help clinicians with the diagnostic process of ACL and PCL injuries.

## Conclusion

This study from the SNKLR shows that patient characteristics and activities at the time of injury resulted in specific injury profiles for ACL and PCL, as follows. (1) Females, older patients and patients with a higher BMI were more susceptible to more complex injuries, such as combined PCL injuries; (2) a larger proportion of combined ACL, meniscus and cartilage injuries was found in females; (3) isolated ACL injuries were most frequently found in males; (4) ACL injuries were sustained during pivoting sports or alpine skiing; (5) PCL injuries were sustained either during pivoting sports, non-pivoting sports or were traffic related. These data provide valuable information to increase our understanding of how specific injury forms of cruciate ligament injury (ACL and PCL) occur and can help clinicians with the diagnostic process of ACL and PCL injuries.
